# The Invisible Burden: Structural and Emotional Barriers to Nutritional Care in Mexican Breast Cancer Patients

**DOI:** 10.3390/healthcare13212817

**Published:** 2025-11-06

**Authors:** Miriam Leticia Guevara-Rangel, Luis Eduardo Hernández-Ibarra, Blanca Alejandra Diaz-Medina, Darío Gaytán-Hernández

**Affiliations:** 1 Faculty of Nursing and Nutrition, Autonomous University of San Luis Potosí, San Luis Potosi 78290, Mexico; 2 Escuela de Medicina, Universidad del Valle de México, Campus Zapopan, Zapopan 45010, Mexico

**Keywords:** breast cancer, comprehensive care, experiences, qualitative research, Mexico

## Abstract

**Background/Objectives:** Breast cancer is one of the most prevalent types of cancer globally and is also the leading cause of death from malignant tumors among women. This study investigated the experiences of women with breast cancer concerning nutritional care. **Methods:** A qualitative study conducted in San Luis Potosí, Mexico. Ten semi-structured interviews were conducted with women undergoing treatment and with survivors of breast cancer. Data were analyzed using basic grounded theory procedures. **Results:** Our findings revealed several barriers that hinder adequate nutritional care for women with breast cancer in Mexico. Key barriers include the structure of the health system, misinformation, the implications of the disease in daily life, medical treatment of the disease, the emotional impact, and changes in their perceived identity. The experiences of individuals with any disease, particularly chronic illnesses such as cancer, affect the implementation and follow-up of nutritional therapy. **Conclusions:** Consideration of these experiences is essential for creating policies and strategies focused on nutritional care that complement the treatment of breast cancer sufferers. Additionally, specialized nutrition personnel should form part of the comprehensive treatment of the disease.

## 1. Introduction

Breast cancer is one of the most prevalent cancer types globally and the leading cause of death from malignant tumors among women. In Mexico, during 2023, there were 834 deaths from breast cancer in the population aged 20 years and older, with a national mortality rate of 17.9 per 100,000 women [1].

Women with cancer represent a population that receives insufficient attention and recognition in the field of nutrition. Breast cancer is a multifactorial disease strongly influenced by diet and lifestyle. The relationship between nutrition and cancer is two-fold, as diet can be both a risk factor and a preventative factor. In postmenopausal women, overweight and obesity increase estrogen production in adipose tissue, which increases the risk of developing cancer. Therefore, prevention focuses on promoting healthy lifestyles and reducing risk factors [2].

During diagnosis, treatment, and recovery, people with cancer face nutritional risk due to the emotional impact that negatively impacts dietary intake and the side effects of treatments. It is estimated that at least half of the women who suffer from or are at risk of developing this condition experience weight loss within the next six months [3]. This weight reduction negatively affects their health status, as malnutrition increases the risk and number of complications during cancer treatment and reduces treatment tolerance, which, in turn, diminishes the patient’s quality of life. Furthermore, there are alterations in body image, self-esteem, muscle strength, physical activity, and social functionality [4].

Conversely, those who complete their treatment may experience nutritional problems as a consequence of the medical procedures used. Often, unwanted weight gain is associated with changes in body composition, characterized by increased fat mass and loss of muscle mass, which raises the risk of developing chronic diseases such as diabetes, osteoporosis, hypercholesterolemia, and cardiovascular disease [5]. Frequently, women who survive cancer do not receive adequate nutritional care in the clinical setting, despite the recognized importance of nutrition among survivors [6].

Individuals with breast cancer face difficulties in maintaining adequate nutrition. These challenges may arise due to the disease and its treatments, affecting their emotional state and lifestyle. In addition, the lack of management strategies by a multidisciplinary team within the health sector and inadequate nutritional planning by nutrition professionals aggravate the situation. It is often overlooked that the act of eating encompasses not only the physical intake of nutrients but also mental and social aspects, influenced by various internal and external factors, such as culture, beliefs, ethical values, income, and individual food preferences and aversions [7].

Consequently, nutritional problems in people with cancer have varied origins, and the benefits of personalized nutritional care throughout the disease process should not be underestimated. Cancer control actions throughout comprehensive care (i.e., prevention, detection, medical care, rehabilitation, and palliative care) interact with the social inequalities prevalent in Mexico. The economic, educational, and insurance conditions of individuals also determine the characteristics of cancer treatment available to those affected.

The implementation of national actions to control and treat cancer faces significant challenges: insufficient services and infrastructure, limited and low-quality screening interventions, and inadequate follow-up of treated patients. These inadequacies result in more than 70% of cancer diagnoses occurring at advanced stages, which reduces the likelihood of a cure and increases treatment costs, estimated at 2500–5000 USD per person with breast cancer in institutions such as the Mexican Social Security Institute, with costs rising to 17,000 USD if the person is treated in a private institution. Indeed, recent data has shown that cancer care protocols in Mexico are often outdated and fail to represent the best standard of care. The international recommendation is to update these protocols every two to three years to ensure the best available treatment [8].

Given this context, we aimed to explore the experiences of women with breast cancer regarding nutritional care.

## 2. Materials and Methods

### 2.1. Setting

We conducted a qualitative study with a constructivist approach, was conducted in the city of San Luis Potosí, Mexico, between October 2023 and March 2025.

### 2.2. Study Design and Participants

Participants were selected through purposive sampling, considering inclusion criteria aligned with the research objective. The final sample consisted of ten women: five volunteers from a civil association and five patients undergoing antineoplastic treatment at a public health institution. Their ages ranged from 41 to 69 years, with an average age of 55 years. More than half of the participants were married at the time of the study; only three of them were paid workers. All participants received treatment through the national health system. The sample size was determined based on the criterion of theoretical saturation, that is, the point at which the information obtained no longer provided new conceptual elements or relevant categories for analysis (Table 1).

### 2.3. Data Collection

Semi-structured interviews, each averaging 60 min, were conducted. An interview guide, developed from a literature review, contained open-ended questions to explore participants’ experiences in seeking nutritional care as complementary management for their condition. The interviews were audio-recorded with informed consent and subsequently transcribed using a pre-established format. Transcriptions were carried out faithfully, preserving the participants’ exact words and the quotes were translated from Spanish with accuracy check to maintain the fidelity of the participants voices. Ellipses were used to indicate instances where conversation occurred before or after the quoted excerpts, without altering the original meaning of the statements. All authors participated in reviewing the materials, coding and identifying themes for categorization, and selecting excerpts from the interviews (Appendix A).

### 2.4. Data Analysis

Constructivist grounded theory procedures were employed for data analysis. Open coding led to the emergence of study categories, which were further developed through focused coding and constant comparison across codes and categories. Emerging categories were refined, compared with the data for fit, and saturated. As analysis progressed, memos were developed to achieve higher conceptual levels. These memos were clustered around the main categories, which were reviewed again for data fit [9]. Study rigor was ensured by focusing on data emergence via open questions in the guide and the interviewees’ answers. Inductive analysis and constant comparison maintained category emergence integrity. A reflective diary was maintained during fieldwork. The first author and the corresponding author independently analyzed the data and participated in all coding. After each coding phase was completed, they met to discuss the various codes and decisions to determine points of agreement and disagreement.

To ensure the study’s rigor, a systematic process was employed, which included: (a) open-ended interview questions to facilitate the emergence of data, (b) inductive analysis with constant comparison for category generation, and (c) continuous verification of the fit between the categories and the data. Furthermore, reflexivity was supported through the use of a fieldwork journal and analytical memos taken during data analysis.

### 2.5. Ethical Considerations

The study adhered to the ethical principles of the Declaration of Helsinki and was approved by the Ethics Committee of the Autonomous University of San Luis Potosí (CEIFEN-2023-425). The participants’ autonomy, confidentiality, and anonymity were respected at all times.

## 3. Results

From the perspective of the participating women, a series of situations arise during the course of the disease, exposing them to experiences originating from structural, emotional, existential, medical, and social challenges. Cancer diagnosis and treatment is a severe stressor capable of triggering multiple physical and mental symptoms that negatively impact a woman’s quality of life [10].

These challenges negatively influence women with breast cancer as they undertake nutritional therapy as a complementary treatment to improve their health condition and reduce the likelihood of recurrence.

### 3.1. Cast Adrift by Illness

The participants reported experiencing various events that induced uncertainty upon learning of their diagnosis, causing initial disorientation that affected their decision-making during treatment. Some of the situations indirectly affecting adherence to nutritional treatment are listed below (Table 2).

#### 3.1.1. Race Against Time

The participants reported experiencing significant time pressure, as cancer is often advanced by the time any sign or symptom is suspected. The impact of the diagnosis, coupled with visualizations of catastrophic scenarios and the rapid commencement of oncologic treatment, compels them to act quickly and sometimes with uncertainty. This fear of death, the perception of having limited time, and misinformation can lead to inappropriate or erroneous decisions, as they may not fully process the situation.

#### 3.1.2. Fighting Alone

Family and support networks are essential for accepting and better coping with the disease, as well as for adhering to prescribed treatments, including nutritional interventions. Relationships with family members are affected during diagnosis and treatment. Participants reported feelings of loneliness and vulnerability, perceiving themselves as different from their family members. The associated negative mood changes generated tension and concern within the family, prompting them to isolate themselves and feel misunderstood or burdensome. This led to the perception of needing to face the disease alone, as they did not want to trouble or worry their families. In this regard, loneliness can exacerbate existing mental health conditions by amplifying feelings of worthlessness and hopelessness. Feeling lonely can also increase vulnerability to stress throughout the illness process [11].

#### 3.1.3. Lack of Information

A shared experience among participants was the lack of information provided by healthcare personnel before, during, and after receiving their diagnosis, as well as the medical treatments. They often felt overwhelmed following the diagnosis, compounded by the limited consultation time. The scarce information was dominated by scientific or technical jargon used by medical staff, and bureaucratic procedures within institutions resulted in low comprehension and assimilation of necessary information, hindering the follow-up of administrative and therapeutic instructions.

#### 3.1.4. Dehumanizing Treatment

Women reported perceiving a lack of empathy from healthcare personnel, as well as a sense of not being involved in their treatments. This led to feelings of receiving impersonal care and created barriers to establishing trust, prompting women to withhold their questions. In response, some sought care in private services due to the long waiting times in public hospitals. They described these private experiences as more pleasant, characterized by more humane and attentive interactions. However, they eventually had to continue their treatment within public institutions because of economic constraints and insurance coverage. Despite this, participants commonly reported that care in the public system felt impersonal, fragmented, and marked by a sense of being ignored or treated routinely, without attention to their individual or emotional context. Consequently, they sought information and strategies elsewhere such as through other women’s experiences, on the Internet, in magazines, or in books.

#### 3.1.5. Prescriptions Beyond Their Means

A significant barrier hindering nutritional changes among women with cancer was the perception of “unattainable” prescriptions. These were primarily associated with three interrelated dimensions. First, affordability, as participants reported financial limitations that made it difficult to pay for the recommended foods or supplements. Second, availability, since they were not always able to access the products or services suggested by health professionals, whether due to limited local supply or logistical challenges. Finally, appropriateness, as some recommendations did not align with their cultural habits, family food preferences, or the treatment side effects that altered appetite and tolerance to certain foods. Although they recognized the expertise of health professionals, they believed that these professionals did not consider each patient’s context, complicating the adherence to nutritional and dietary prescriptions alongside medical treatment.

The uncertainty present throughout the journey of those suffering from this disease is the principal source of various emotions, such as fear and concerns about the future. The main concerns of participants included disclosing their condition to family and the fate of their children should they lose their lives to cancer in the near term. Acting strongly in front of others while uncertain how to maintain pre-diagnosis routines creates a barrier to adapting to new routines.

### 3.2. Not Being the Same as Before

The emotional impact experienced by women with breast cancer can negatively affect their social, family, and work life. The altered perception of their self-image as women presents challenges throughout the disease and even thereafter, influencing their decision-making regarding treatment adherence and the pursuit of life’s meaning. Cancer poses a personal challenge, and women often face several hurdles (Table 3).

#### 3.2.1. Facing a New Reality and Not Recognizing Oneself

Facing an illness means entering an unfamiliar world that affects daily life, causes changes in routine and body image, and introduces new habits. Medical treatments for this disease challenge women’s identity, self-esteem, and body image perception. Many women experienced mood declines and even depression following hair loss due to chemotherapy or mastectomy, which altered their appearance drastically. Such sudden, unwanted changes led to low self-esteem, feelings of stigmatization, and comparisons with other women, which could increase depression or prompt the search for accessories and personal adornment (e.g., rings, necklaces, etc.) to feel reintegrated into society. Low self-esteem has been associated with greater psychological distress and poorer adherence to treatment. Low self-esteem has also been linked to being a risk factor for depression and social isolation [12].

Chemotherapy is an especially abrasive treatment. The type, dose, and frequency lead participants to feel intoxicated, as it alters their senses of taste and smell, physical appearance, and perception of hunger, often accompanied by sweating out the medication. These experiences fostered a perceived need to “cleanse or purify” themselves by increasing water intake or opting for more natural foods.

#### 3.2.2. Grief

Disease-related health loss triggered symptoms related to the loss such as an altered sense of identity, intense emotional pain and emotional numbness [13], which were reflected in feelings of sadness, frustration, fear, anguish, anxiety, perceived as an unfair affliction occurring without warning. Depending on the prognosis and treatment challenges, feelings of hopelessness and low mood prevailed. During the grief experience induced by disease, denial, and fear dominated. Participants feared bad news, struggled to accept and verbalize the illness, feared medical visits and test results, and faced uncertainty on treatment initiation. Coupled with relatives’ and friends’ remarks, this created overwhelming negative thoughts.

#### 3.2.3. Feeling Like a Burden

This process altered physical conditions, causing fatigue, restricting daily activities, and necessitating help for basic tasks, disrupting family routines. Especially after treatment sessions, participants felt burdensome due to changes in roles or inability to perform familiar tasks.

### 3.3. Staying Afloat

Cancer can leave both physical and emotional scars. For the women in this study, the degree of adaptation to cope with difficult situations varied and was influenced by previous experiences with illness either personally or in close relatives, shaping their survival outlook. Nonetheless, we found that the participants employed several strategies to stay afloat amidst the disease, as outlined below (Table 4).

#### 3.3.1. Facing Adversity

The desire to persevere stimulated the search for ways to manage treatment-related discomforts. Women adapted strategies according to their capabilities and close information sources, including researching online, reading books, and connecting with other women undergoing similar treatments through supportive associations, sharing experiences, and economic support. Focusing on finding solutions reduces stress, which helps empower women in the face of successive challenging situations, allowing them to improve their self-concept [10].

#### 3.3.2. Adopting a New Perspective

Experiencing a life-threatening disease led some women to view this ordeal as a life lesson, prompting healthier lifestyle adoption. This shift occasionally prompted drastic habit changes previously unrecognized as harmful. Seeking healthier living strategies extended beyond themselves to prevent similar familial or social situations.

#### 3.3.3. Turning Experience into Testimony

Having overcome a difficult experience felt akin to an achievement to the women involved in this study. By sharing their experiences and advice, they aimed to aid those beginning or undergoing similar processes, offering gratitude to those who aided them by sharing strategies to navigate their own challenges.

## 4. Discussion

We aimed to explore the experiences of women with breast cancer in seeking nutritional care and identified multiple barriers that hinder adequate nutritional care for these women in Mexico. The key challenges include the structure of the Mexican healthcare system and psychosocial factors experienced by women during their illness.

This research delineated three categories to explain women’s experiences in seeking nutritional care from their perspective. Throughout the participants’ discourse, misinformation emerged as a critical factor impacting them and their families, leading to negative emotions such as distress, uncertainty, time pressure, sadness, and anger. This misinformation was evident at the time of diagnosis, as the meaning of cancer, its life implications, and available treatments were often unknown.

Having enough information is crucial for individuals with any disease, particularly chronic diseases. Research has shown that increased knowledge contributes to better management of treatments and improved physical and mental health, demonstrating a clear relationship between disease perception and quality of life [14].

Within family dynamics, positive aspects include unity, coexistence, companionship, household task performance, and childcare. Conversely, negative aspects involve haste, overprotection, lack of support, and feelings of burdening family members, which strain relationships between partners, children, siblings, and elderly parents, causing tension and separation between the ill and healthy family members [15].

These findings corroborate the literature indicating that a cancer diagnosis is often perceived as a negative, catastrophic life crisis due to its association with suffering and death. These perceptions lead to anxiety, reactive depression, and isolation tendencies. The idea of death prompts fears of separation from loved ones and abandoning life goals [16].

A common experience among women undergoing antineoplastic treatment is gastrointestinal disturbance, affecting appetite and altering relationships with food. This leads to the avoidance of foods that exacerbate symptoms or are feared due to associations with cancer, including meats, fats, and sugar [17].

Contributing factors include a lack of nutritional guidance on the role and benefits of nutritionists, physicians’ failure to recommend nutritionists, and financial barriers to accessing nutritional therapy. The Mexican public health system has limited nutrition staff, who often manage multiple hospital areas. Health professionals attempt to address these deficiencies by utilizing students in training, such as interns who lack the requisite knowledge and skills to care for complex or specialized diseases [18].

The relationship between nutrition personnel and hospital patients is strained due to overburdened health professionals and standardized prescriptions with fixed consultation times. This scenario alters the delivery of specialized, individualized care, leading physicians to refer women with cancer to nutritionists only in critical health situations. Consequently, patients resort to private services, incurring additional expenses, and forcing a choice between medical treatments and nutritional consultation [18].

When discussing the incorporation of new eating habits and lifestyles, it is crucial to evaluate the relevance and cultural impact of interventions designed for chronically ill individuals. These actions and prescriptions extend beyond merely alleviating certain symptoms and signs of disease, as they affect both the family and the broader community.

Social support acts as a protective factor for women with cancer and helps reduce the intensity of effects and changes associated with the disease, facilitates adaptation to these changes, and assists with crisis management. Moreover, it accelerates recovery and enhances treatment adherence [19].

Dietary changes can create a division within a family between those who are sick and those who are not, leading to a disruption in familial coexistence. Furthermore, managing oncological treatment is often financially burdensome, and accessing nutritional care can be prohibitively expensive, sometimes even viewed as exotic since the recommended foods or products may be beyond their economic reach [20].

In the category “Not being the same as before,” we attempted to include all the emotions the women reported experiencing during the disease and that directly and indirectly alter their self-perception, views of the environment, and relationships with the process of accepting and overcoming the illness.

The emotional impact associated with cancer can serve as a trigger or a facilitator during the disease’s progression or a potential relapse. This impact is influenced by the type and degree of personal discomfort, which are themselves modified by the disease stage, prescribed treatments, age, personality variables, and social support. Women can experience positive emotions, such as hope and gratitude. However, many women may have more difficulty with negative emotions, such as anxiety, sadness, anger, guilt, and fear. While all of these emotions can be common reactions to the diagnosis, negative emotions can range from unpleasant to debilitating [21].

There is evidence that psychological distress, especially depression, leads to a worse prognosis in cancer patients. Cancer has complex consequences in various areas of life, including psychological, social, familial, and economic impacts [22].

Economic difficulties arising from the disease are a significant source of distress, as they often result from interruptions in employment for the patient or the family caregiver, potentially leading to a decline in daily activities, loss of life’s meaning, and negative emotional states [23]. The primary factors influencing the emotional impact at the time of a cancer diagnosis include limited or inadequate knowledge of cancer, previous personal experiences with cancer through family or friends, excessive fear, feelings of guilt and fatalism, and a sense of vulnerability to the disease.

Among the emotions reported by the participants, many were associated with a change in self-identity, such as not recognizing oneself, feeling burdened by medication side effects, and perceiving oneself as a burden to family members. This often generates anxiety about leaving family members prematurely in case of complications. Addressing challenges related to identity, self-esteem, and body image perception in women begins with understanding the implications of the tumor’s location, as it often involves intimate organs associated with maternity, femininity, and sexuality.

Emotional state alterations directly influence health by affecting physical functioning, symptom recognition, timely care-seeking, and engaging in healthy or unhealthy behaviors. Negative moods increase vulnerability to infectious diseases, elevate symptom perception and reporting, and reduce medical or nutritional care-seeking [24]. Negative emotional states (e.g., depression and anxiety) can impede healthy behaviors and instead trigger harmful ones or diminish patients’ confidence in self-care practices. In fact, it is not merely the emotional response influencing healthy habits but rather the cognitive element of worry that motivates individuals to adopt a healthy lifestyle [14].

The third category, “Staying afloat,” covered the strategies used for confronting and overcoming the disease. Cancer must be viewed as a continuous process; after treatment concludes, the journey continues with ongoing medication, which has less physiological impact, and constant monitoring, which varies in duration based on breast cancer type, progression before diagnosis, and prior treatment. This ongoing process also emotionally impacts individuals, who must relive difficult moments and face fears of potential relapse [25].

Women strive to rebuild hope and find meaning in life through their experiences, viewing the disease process as a teaching or life lesson. The common sentiment among the interviewed women was to enjoy what makes them happy and share their experiences to assist others in similar situations. People often create self-care tools to ease the burden of their situation [26]. One strategy involves using life stories to recreate and represent the essence of their experience, turning experiences into testimonies. This allows for re-signification of their experiences and response to a newly explored world, offering a moment to rethink values, beliefs, and thoughts, potentially leading to growth and change.

Contrary to Charmaz (1995) [27], who suggested chronic illness might cause a loss of self, a reduction in crucial aspects defining identity, this study found that women who shared their experiences found a purpose, encouraging their progress. As part of information search and testimony creation, women with cancer often approached associations for emotional and humanitarian support, typically lacking in biomedical contexts. Many association volunteers have experienced cancer and chose to continue helping more women [8,27].

## 5. Conclusions

This qualitative study illuminates the profound and multifaceted barriers that women with breast cancer in Mexico face when seeking nutritional care. Our findings underscore that these challenges are not merely clinical but are deeply rooted in structural deficiencies of the healthcare system, socioeconomic disparities, and the profound psychosocial impact of the disease. The experiences of participants—characterized by dehumanizing treatment, unattainable prescriptions, a lack of information, and a disrupted sense of self—collectively highlight a critical gap in the standard of comprehensive cancer care.

The consistent absence of integrated, specialized nutritional guidance within oncology protocols exacerbates patient vulnerability, potentially worsening treatment outcomes, quality of life, and long-term survival. Therefore, it is imperative that healthcare policies in Mexico recognize nutritional care not as an ancillary service but as a fundamental pillar of oncological treatment. This necessitates the formal inclusion of registered dietitians and nutritionists within multidisciplinary oncology teams from diagnosis through survivorship.

For future research, this study points to several critical pathways. First, quantitative studies are needed to measure the prevalence of these barriers and their correlation with clinical outcomes. Second, investigating the perspectives of healthcare providers, administrators, and policymakers is crucial to understanding the systemic constraints and developing feasible solutions. Finally, intervention studies focused on developing and testing culturally appropriate, economically viable nutritional support models are essential.

This study has limitations. The findings, while rich in detail, are derived from a specific sample of women in San Luis Potosí and their experiences within a particular timeframe. Consequently, they may not be fully representative of all healthcare contexts or the diverse population of women with breast cancer across Mexico. The qualitative nature of this research prioritizes depth of understanding over generalizability.

In summary, addressing the nutritional care of women with breast cancer demands a paradigm shift towards a more humane, personalized, and integrated model of care. By centering the experiences of patients and dismantling the structural barriers they encounter, we can move closer to an equitable healthcare system that truly supports the journey towards healing and survival.

## Figures and Tables

**Table 1:** Characteristics of the study participants.

Code	Age	Occupation	Marital Status	Years with the Disease	Type of Treatment	Healthcare System *
P1	41	Government employee	Divorced	6	Chemotherapy Radiation therapy	Private healthcare
P2	56	Housewife	Married	6	Surgery	Private healthcare
P3	60	Housewife	Single	Survivor	Surgery Chemotherapy Radiation therapy	IMSS Bienestar
P4	53	Retired/Housewife	Divorced	6	Surgery Chemotherapy	ISSSTE
P5	58	Teacher	Married	Survivor	Surgery Chemotherapy	IMSS
P6	69	Housewife	Married	Survivor	Surgery Chemotherapy	ISSSTE
P7	66	Non-medical caregiver	Widow	17	Surgery Chemotherapy Immunotherapy	IMSS Bienestar
P8	50	Homemaker	Domestic partnership	4	Chemotherapy Radiotherapy	IMSS Bienestar
P9	52	Housewife	Married	17	Chemotherapy	IMSS Bienestar
P10	49	Housewife	Married	2	Chemotherapy	IMSS Bienestar

* IMSS (Instituto Mexicano del Seguro Social) and ISSSTE (Instituto de Seguridad y Servicios Sociales de los Trabajadores del Estado) are public healthcare institutions in Mexico. IMSS Bienestar is a branch of IMSS that provides medical services to uninsured and underserved populations.

**Table 2:** Cast Adrift by Illness: Excerpts from women with breast cancer regarding isolation and systemic neglect.

Subcategory	Interview Excerpts
Race against time	“In cancer, life is slipping away from you, slipping away so quickly, and it passes by rapidly.” (P3) “If I had waited longer, it could have started to metastasize, and I wouldn’t be here today.” (P2) “I had surgery privately precisely because of the urgency, because if I had gone through the service I have at ISSSTE, it would have taken much longer.” (P4)
Fighting alone	“I started crying by myself, I wish someone was with me […] I didn’t feel any emotional support from him [her significant other], I had to fight alone.” (P8)
Lack of information	“I had just received the news and didn’t know what cancer was, since I knew very little about it, and I got really nervous.” (P9) “I would like there to be guidance regarding nutrition and all the aspects one goes through during chemotherapy. They vary, but they could be unified in terms of how things should be done.” (P6) “I didn’t have enough knowledge to manage the care needed.” (P6)
Dehumanizing treatment	“I would ask him, ‘Doctor, I just feel so weak. What can I do?’ One day, the doctor from the public clinic told me, ‘Well, what do you want me to do? That’s how it feels. What do you want? You want me to prescribe you 1 kg of vitamins? Fine, I’ll do it.’ And I said, ‘It’s not about answering me like that [*sic*], why not just tell me what I can eat so I can feel better?’ [And he said] ‘Ugh, just eat like five walnuts, or five almonds, if you can afford them.’ That’s how he spoke to me. And sometimes, I didn’t even have money, but my sister would come along and say, ‘Here, so you can have your little treat.’ I couldn’t [afford to] eat them every day, of course not, but once a week, I knew it helped me. I asked, ‘Are you going to refer me to a nutritionist?’ And he said, ‘What for? Everything’s just going to make you feel nauseous anyway.’” (P3) “She just handed me a little sheet of paper and said, ‘Well, since they removed some foods from your diet, just cut out anything animal-based, and for the rest, follow these portions every day.’ And that was it. From there, it was like ‘figure it out yourself’—add and subtract portions however you can. I was really looking for someone who actually cared about me as a patient.” (P4)
Prescriptions beyond their means	“She might be the best nutritionist and have the best program, but how do you bring that to a population where, for the most part […] it’s already hard enough just going through cancer treatment, and then you still need to match it with an excellent nutrition plan.” (P4) “There are so many factors a nutritionist has to consider in these cases, [like] people’s social class [and] their habits.” (P4) “Nutritional care should be more specific. And in my case, honestly, I just didn’t find the help I was hoping for.” (P4) “They provide you a list of food that I simply cannot afford.” (P9)

**Table 3:** Not Being the Same as Before: Excerpts from women with breast cancer regarding identity disruption and emotional burden.

Subcategory	Interview Excerpts
Facing a new reality and not being the same as before	“For me, it was three major losses: my health, my partner, my home, and my business [*sic*]. My health had already been deteriorating, but the losses were caused by the cancer.” (P3) “The hardest part is having to change your routines, because you’re already so used to them.” (P1) “Yeah, she started telling me all this information, and instead of helping, it overwhelmed me. My mind was in total chaos.” (P4) “It wasn’t my breast anymore, it was just a chunk [of meat], and the rest was swollen tissue. I looked at it and said, ‘What is this?’ I didn’t understand. I mean, why leave it like that if I’m going to end up deformed anyway?” (P3) “How is it possible that now my legs won’t support me, and when they were fine, I never appreciated them? And now I desperately need them just to stand up.” (P3)
Grief	“It’s like a personal kind of grief, because there’s no one you can blame, no one to say, ‘I hate you’ to.” (P5) “‘Warrior’? I didn’t want to fight anything. I was perfectly at peace where I was. What warrior? No! We’re not warriors, and none of us ever wished for this.” (P4) “I’ve come to realize there are no words that really lift you up to keep going. You can’t skip the process, because it will hit you either way.” (P7)
Feeling like a burden	“I had always lived alone and been independent. Then I got sick and had to move in with my relatives because I couldn’t manage on my own. They had to drive me places and pick me up after. I couldn’t even go near the stove, so they had to cook everything for me.” (P3) “They told me, ‘Yeah, things have changed because all of a sudden you can’t even go out with us anymore.’” (P8)

**Table 4:** Staying Afloat: Excerpts from women with breast cancer regarding growth and shared experiences.

Subcategory	Interview Excerpts
Facing adversity	“…So when you’re in the middle of it, you start looking for what might help. Like, someone says *quelites* [plants used in traditional cuisine] are good. Well, I don’t even like them, but if they say they help, I’ll eat them. I started looking things up online, and you then you make friends with other patients who might say, ‘Hey, what works for you?’ ‘Oh, well, I eat chicken soup…” (P7) “…You have to adapt the illness to your situation, and it is possible. It just takes willingness, research, and effort, and you’ll get through it…” (P5) “…So, for her [the nutritionist], it was really hard to come up with a diet plan for me. After that, I stopped seeking nutritional support. Instead, I started browsing online and learning about other kinds of foods that I might actually like…” (P4)
Adopting a new perspective	“After cancer, or after something like that, you honestly want to take on the whole world. You want to enjoy it all. You see things differently. Life feels different. For me, honestly, breast cancer was a blessing. It brought blessings pouring into my life. It was a huge turning point. I was one person before and a completely different one after. For me, it was this incredible opportunity to really live, to rediscover myself, to reflect on everything, to learn from it, and I want to keep learning more.” (P4)
Turning experience into testimony	“If I can be a helping hand to someone, then that’s great. Even if it’s just for a moment, it’s nice being there for someone because I know now what it feels like to be in their situation.” (P7) “To research [about cancer] so that I can pass it on to others going through the same thing but using my own experience.” (P6)

## Data Availability

The data presented in this study are available on request from the corresponding author due to privacy and ethical restrictions. The datasets presented in this article are not readily available because the primary data collection method for this qualitative study was through interviews. The study participants agreed to participate after receiving assurances, in their informed consent, that the data and their stories would be protected to safeguard their confidentiality and anonymity, including audio-recorded interviews.

## Appendix A. Interview Guide Applied for the Research: “Experiences of Women with Breast Cancer in Seeking Nutritional Care”

**SEMI-STRUCTURED INTERVIEW GUIDE FOR WOMEN WITH BREAST CANCER: EXPERIENCES IN SEEKING NUTRITIONAL CARE—UASLP**

**1. Introduction:**

Good morning/afternoon. How are you? I am Miriam Guevara, a student at the Autonomous University, and I am conducting research aimed at understanding the experiences of women who have breast cancer or are survivors, to learn about their journey in seeking nutritional care as well as their experience during nutritional treatment. The interview will last approximately one hour. The information you provide will be confidential and used solely for the purposes of this research. Your participation is voluntary; you have the right to take part or not in the interview.

If you agree to participate, we will talk about some general information and also about your life, your health, and how you are experiencing this stage of your life. In addition, I will ask for your permission to record the interview and take some notes in order to accurately capture the information you provide. You do not have to answer any question you do not wish to, and you may stop the interview at any moment. Also, if at any point during the interview you have questions or need a break, feel free to let me know.

Do you have any questions?

Would you like to participate?

May we begin the interview?

Do you agree to have the interview audio recorded?

**2. Let’s start by talking about some general information to get to know you a little better:**

**2.1 General Information**

2.1.1 Can you tell me your name? (First name only, to address you personally)

2.1.2 How old are you?

2.1.3 What is your marital status?

2.1.4 What is the highest level of education you have completed?

2.1.5 What is your occupation? (Explore what they spend most of their time on and whether they are still working and why)

**3. Now let’s briefly talk about your experience accessing health services:**

**3.1 Access to Health Services**

3.1.1 Do you currently have access to any health service, and if so, which one? (Clarify whether it is public, private, or job-related)

(If yes, identify the type of service and through whom they have it. If no, identify where they seek care when ill and how they pay for it.)

3.1.2 When you get sick or feel unwell, where or with whom do you seek care?

(Explore whether they use only one health service or multiple: public, private, alternative medicine, etc.)

3.1.3 How do you get there? Do you go alone or with someone? Who accompanies you?

**4. Now we’ll talk about your experiences related to your illness:**

**4.1 Current health condition, experiences with treatments, known cases, family history (these questions are more sensitive and richer in information):**

4.1.1 What has your experience been like with the healthcare professionals who attend to you during your treatment?

4.1.2 What kinds of medications or treatments are you receiving for your condition?

4.1.3 How do you obtain or pay for these medications or treatments?

4.1.4 Could you tell me if someone helps you with your care, and what that support or care consists of?

4.1.5 What is your current diet like? Could you share the most important changes you’ve noticed?

4.1.6 What do you do to take care of yourself and feel better? (Explore if they attend support groups or if they carry out individual activities related to diet, physical activity, etc.)

**4.2 Sociological, psychological aspects, social determinants of health, etc. = barriers to overcome =**

4.2.1 Could you tell me how you found out about your illness?

4.2.2 What changes have you noticed since then?

4.2.3 What has been the most difficult part and why?

4.2.4 What advice or recommendations would you give to others going through a similar process?

**5. Now let’s talk about your diet…**

**5.1 Nutrition-Specific Questions**

5.1.1 How did your diet change when the illness began?

5.1.2 Were you referred to or did you seek out a nutrition professional? What were the reasons? (Explore motivations, beliefs, perceptions)

5.1.3 Tell me what your visits to a nutrition consultation were like: Who accompanied you? Where did you go? How long did you have to wait?

5.1.4 What would you say is the most difficult part of following a nutritional treatment, and why?

5.1.5 What changes have you made since receiving nutritional care, and how have you felt?

5.1.6 In your opinion, what aspects of health and nutritional care services could be improved?

**6. Thank you very much! We have finished.**

- Is there anything else you would like to add?
- Is there something you consider important that I didn’t ask?
- If necessary, would it be okay to contact you again to clarify any points?

**THANK YOU VERY MUCH FOR YOUR TIME AND GENEROSITY.**

## References

[1] INEGI Estadísticas a Propósito del día Internacional de la Lucha Contra el Cáncer de Mama (19 de Octubre). 2024. https://www.inegi.org.mx/app/saladeprensa/noticia/9346.

[2] López-Blázquez L., Ramiro-Armuña I. (2018). Influencia del estado nutricional en la prevención y evolución del cáncer de mama. Nutr. Clín. Diet. Hosp..

[3] Cancer E., Orúe I., Estornell M., Sánchez-Sánchez E., Guerra J., Barragán B., Blanco M., De Paz H. (2022). Opinions and experiences of healthcare professionals in the nutritional management of oncology patients: The ONA study. Nutr. Hosp..

[4] Salamanca S., Perez J., Acevedo E. (2020). Experiencias personales y profesionales de pacientes con cáncer de mama adscritas a un centro de oncología de Santander. Inf. Psicol..

[5] De Cicco P., Catani M., Gasperi V., Sibilano M., Quaglietta M., Savini I. (2019). Nutrition and Breast Cancer: A Literature Review on Prevention, Treatment and Recurrence. Nutrients.

[6] Sullivan E., Rice N., Kingston E., Kelly A., Reynolds J., Feighan J., Power D., Ryan A. (2021). A National Survey of Oncology Survivors Examining Nutrition Attitudes, Problems and Behaviours, and Access to Dietetic Care Throughout the Cancer Journey. Clin. Nutr. ESPEN.

[7] Gómez M., Marín S., Benítez V., Loria M., García T., Lourenco M., Villarino R., Castillo R., Zamora P. (2008). Autopercepción de los pacientes con cáncer sobre la relación existente entre su estado nutricional, su alimentación y la enfermedad que padecen. Nutr. Hosp..

[8] Flamand L., Moreno C., Arriaga R., Cetina L., Jiménez M., Arrieta O., Rivera S., Reyes H. (2021). Cáncer y Desigualdades Sociales en México 2020.

[9] Strauss A., Corbin J. (2016). Bases de la Investigación Cualitativa: Técnicas y Procedimientos Para Desarrollar la Teoría Fundamentada.

[10] Argelo-Fernández A., Fernández-Arce L., Llaneza-Folgueras A., Encinas-Muñiz A., Olivo M., Lana A. (2025). Coping Strategies and Health-Related Quality of Life in Breast Cancer Survivors. Eur. J. Investig. Health Psychol. Educ..

[11] Egaña-Marcos E., Collantes E., Diez-Solinska A., Azkona G. (2025). The Influence of Loneliness, Social Support and Income on Mental Well-Being. Eur. J. Investig. Health Psychol. Educ..

[12] Rat L., Ghitea T., Maghiar A. (2025). Effectiveness of a Self-Esteem Enhancement Intervention Integrated into Standard CBT Protocol for Improving Quality of Life in Patients with Colorectal Cancer. Eur. J. Investig. Health Psychol. Educ..

[13] Sardella A., Musetti A., Caponnetto P., Quattropani M., Lenzo V. (2023). Prolonged Grief Disorder and Symptoms of Anxiety and Depression among Bereaved Family Caregivers in the Context of Palliative Home Care. Eur. J. Investig. Health Psychol. Educ..

[14] Lemos M., Torres S., Jaramillo I., Gómez P., Barbosa A. (2019). Percepciones de la enfermedad y hábitos de vida saludable en personas con enfermedades crónicas. Psicogente.

[15] Altuve J. (2022). La Familia y el Cáncer: Significados Construidos a Partir de Sus Vivencias.

[16] Parra C., García L., Insuanity J. (2011). Experiencias de vida en mujeres con cáncer de mama en quimioterapia. Rev. Colomb. Psiquiatr..

[17] Mestre G., Moyta M., Velázquez A., Jiménez M., López F. (2013). Nutrición Oncológica.

[18] Rosas N., Hernández-Ibarra L., Vestena J., Rangel Y., Gaytán D. (2020). Structural Barriers in Care Nutrition for People with Chronic Kidney Disease in Mexico. Saúde Soc..

[19] Gil A. (2022). Apoyo Social Percibido y Calidad de Vida en Mujeres con Cáncer de Mama.

[20] Castro A. (2007). La nutrición como ruptura cultural: La experiencia de los adultos con diabetes mellitus tipo 2. Investig. Salud.

[21] Conley C., Bishop B., Andersen B. (2016). Emotions and Emotion Regulation in Breast Cancer Survivorship. Healthcare.

[22] Villoria E., Lara L., Salcedo R. (2021). Frequency of Depression and Anxiety in a Group of 623 Patients with Cancer. Rev. Méd. Chile.

[23] Ko E., Nguyen-Grozavu F., Valadez Galindo A. (2023). “I Had to Do It All Alone”: Hispanic Perspectives on Navigating Breast Cancer Treatment. Int. J. Environ. Res. Public Health.

[24] Hernández F., Landero R. (2014). Aspectos psicosociales relacionados con la calidad de vida en mujeres con cáncer de mama. Summa Psicol. UST.

[25] Vivar C. (2012). Impacto psicosocial del cáncer de mama en la etapa de larga supervivencia: Propuesta de un plan de cuidados integral para supervivientes. Aten. Primaria.

[26] Schneider J., Pizzinato A., Calderón M. (2015). Mujeres con cáncer de mama: Apoyo social y autocuidado percibido. Rev. Psicol..

[27] Charmaz K. (1995). The Body, Identity, and Self: Adapting to Impairment. Sociol. Q..

